# Warr;or21: A 21-Day Program to Enhance First Responder Resilience and Mental Health

**DOI:** 10.3389/fpsyg.2020.02078

**Published:** 2020-09-08

**Authors:** Jeff Thompson, Jacqueline M. Drew

**Affiliations:** ^1^Molecular Imaging and Neuropathology Division, Department of Psychiatry, Columbia University Irving Medical Center, New York, NY, United States; ^2^Griffith Criminology Institute, Griffith University, Mt Gravatt, QLD, Australia

**Keywords:** resilience, first responder, mental health, gratitude, law enforcement, mindfulness

## Abstract

First responders face multiple stressors on a daily basis. They have experienced higher rates of anxiety disorders, depression, burnout, post-traumatic stress disorder (PTSD), suicide (Asmundson and Stapleton, 2008), alcohol and substance abuse (Ballenger et al., 2010), and deficient sleep hygiene (Pearsall, 2012) compared to the general population. Existing resilience research can be utilized and adapted to help first responders cope in a positive manner as a form of prevention and also as part of their recovery. New resiliency programs continue to emerge and this paper details one – warr;or21. The warr;or21 program is explained and based on an evaluation of the program’s preliminary data, the results are promising with how the program can assist first responders (and the general public) increase their resiliency and mental health.

## Introduction

First responders face multiple stressors on a daily basis. For current law-enforcement officers, the National Fraternal Order of Police has described the present conditions as more stressful and difficult than it has been in more than 20 years (Kaplan, 2019). For a variety of reasons and causes, including the lack of coping strategies to deal with these stressors, first responders have experienced higher rates of anxiety disorders, depression, burnout, post-traumatic stress disorder (PTSD), suicide (Asmundson and Stapleton, 2008), alcohol and substance abuse (Ballenger et al., 2010), and deficient sleep hygiene (Pearsall, 2012) compared to the general population. Each of these mental health conditions and stressors is the result of complex historical, environmental, and mental factors. Because there is no single cause for each, there certainly is no single remedy or quick-fix solution.

However, despite this reality, we should not concede that this is acceptable or that these conditions are inevitable. Nor should there be an expectation that this situation needs to continue without efforts to enhance prevention and/or recovery. Fortunately, there is ample evidence in neuroscience, resilience, and mental health studies that certain practices can help people to develop greater mental strength, increased calmness, a greater sense of purpose, more connection with others, more realistic and positive outlooks on their lives, greater emotional control, and the ability to reappraise the situations they encounter to acknowledge the positive aspects of each, especially if the situations are ostensibly negative (Reivich and Shatte, 2003; Ochsner et al., 2009; Bonanno and Pat-Horenczyk, 2011; Korb, 2015; Hanson, 2016; Martin and Ochsner, 2016; Hanson and Hanson, 2018; Margolis and Stoltz, 2018; Southwick and Charney, 2018).

This paper evaluates and outlines one specific approach to building first responder resilience and mental health. The first author developed the warr;or21 program specifically for first responders, and it has since become available to, and practiced by, the general public. The program, which will be discussed in more later in this paper, utilizes research in neuroscience, positive psychology, and resilience to provide first responders with daily practices over the course of 21 days that are practical and that specifically address the work they do. Each practice, including controlled breathing and gratitude, is evidence-based drawing from relevant research. The researchers are still collecting data on this specific program and its impact and as such, the program is still in the pilot stage. However, given that, the foundations of the practices and core concept of the program are science-based and the potential benefits of such a program are promising.

## The Cause For Concern: Stress, Trauma, Mental Health Conditions, and Suicide

It is clear that the current situation for first responders is dire; they repeatedly experience severe trauma (Violanti, 2018). To this extent the NYPD’s Police Commissioner has declared a mental health crisis (Madani, 2019). For the purpose of this paper, first responders include police officers, firefighters, and paramedics.

Compared to the general public, first responders face an increased risk of distress, worry, sleep issues, difficulties in concentrating, adverse effects on personal relationships, increases in substance use, and depression (Benedek et al., 2007). Depression and PTSD can be five times higher than that in the general population (Heyman et al., 2018; Substance Abuse and Mental Health Services Administration, 2018). Further, research has connected law enforcement with higher rates of alcohol abuse (for example, see Ballenger et al., 2010) and poor sleep hygiene (Fekedulegn et al., 2016), with some research stating that sleep issues in law enforcement are twice as prevalent as in the general population (Pearsall, 2012). In one study, nearly half (44.5%) of Canadian first responders screened positive for clinically significant clusters consistent with one or more mental disorders (Mellow, 2017).

PTSD affects between 15 and 30% of first responders (van Dam et al., 2010), and it is important to note that approximately 20% of those diagnosed with PTSD also have a substance use disorder. With respect to the connection between PTSD and substance abuse with firefighters, researchers described the rates, when compared to the general public as disturbing (Henderson et al., 2016). The stressors police officers faced are varied, including the inherent danger and risk of being a first responder, the organization for which they work, and a lack of organization support (Violanti et al., 2016). On average, police officers respond to more than 180 traumatic incidents over the course of their careers (Heyman et al., 2018). The stressors first responders face is not limited to those arising from engaging the public. Coghlan stated that there are four categories of police officer stressors: occupational, operational, organizational, and situational (cited in Kaplan, 2019). Over an extended period, stressors and traumatic experiences can take a toll. Mental and emotional fatigue was a factor in 28% of absenteeism within police organizations (Mental Health Commission of Canada, 2014).

Due to the various stressors and mental health conditions that are associated with law enforcement work, police officers have an elevated risk of death due to cardiovascular disease, metabolic syndromes, and various cancers (Violanti et al., 2013). The same study also found that officers’ life expectancy is shorter than the general public. The study demonstrated that the years of potential loss of life for an officer was 21 times larger than the general population. Further, the impact of work stress infiltrates the personal lives of first responders in a negative way (Violanti et al., 2017).

Reliable scientific data are sparse regarding first responder suicide rates, and Heyman et al. (2018) stated that only 40% of firefighter suicides are reported. Despite this limitation, reports have shown that more law-enforcement officers have died by suicide in recent years in the United States compared to those that have died in the line of duty (Blue Help, 2019). According to statistics, there have been 224 United States-based officer suicides compared to 128 officer line-of-duty fatalities (Blue Help, 2019).

Other reports have shown that emergency medical services (EMS) personnel tend to think more about suicide and to attempt it more compared to the general population (Substance Abuse and Mental Health Services Administration, 2018). Research has shown that first responder personnel with both firefighter and EMS duties had a 6-fold increase in reporting suicide attempts over those solely having firefighting duties (Stanley et al., 2016). Finally, in another study, approximately 37% of fire and EMS staff contemplated suicide (Abbot et al., 2015), putting them overall at a higher risk of suicide than the general public (Henderson et al., 2016).

## Stigma

A report by the National Fraternal Order of Police in collaboration with NBC New York drew on nearly 8,000 responses from police officers across the United States (Fraternal Order of Police, 2018). Approximately 90% of the respondents stated that stigma was a barrier to seeking treatment. Further adding to the stigma, more than 90% of the respondents believed that the public and the law-enforcement community lacked awareness that critical stress is a problem in law enforcement. The stigma associated with seeking help is not limited to United States law enforcement or first responders, as it similarly impacts those in Canada, Australia (Australian Federal Police, 2018), New Zealand (Peach, 2019), and the United Kingdom (Stuart, 2017). While some research on stigma in first responder groups has been undertaken more research and data are needed to fully understand its relationship with help-seeking, coping, and health outcomes (Henderson et al., 2016).

## Current First Responder Resiliency Training

One approach that has been discussed in the literature (Spence, 2017) and that has been undertaken within police agencies to tackle mental health issues is the development of resiliency programs. With respect to developing resiliency programs in police departments, first responder agencies must understand personal resiliency (Hesketh, 2018a). Hesketh (2018a) concludes that it is necessary to improve data collection to make resilience practices more effective. Yet it is also clear that organizations can contribute to the promotion of personal resiliency through training and through creating an environment in which leaders encourage their personnel to make resiliency practices part of their daily routines (Hesketh et al., 2015; Hesketh, 2018b).

Although limited in scope and depth, research on building resilience and positive mental health with first responders has shown some promising results. According to McCraty and Atkinson (2012),

“The data suggest that training in resilience building and self-regulation skills could significantly benefit police organizations by improving judgment and decision making and decreasing the frequency of on-the-job driving accidents and the use of excessive force in high-stress situations. Potential outcomes include fewer citizens’ complaints, fewer lawsuits, decreased organizational liabilities, and increased community safety.”

Beyond the benefits of an agency conducting this training, it clearly (and importantly) benefits the individual’s well-being.

Preliminary evaluations of training used in Australia and Canada, *Road to Mental Readiness (R2MR)*, has shown promising results, with participants stating that it helps to reduce stigma and to build resiliency. They also found that the training was applicable in both their work and their personal lives (Mental Health Commission of Canada, 2014). A study in the San Diego Police Department (Weltman et al., 2014), although based on a small sample (*n* = 14), found that resilience practices yielded positive results. The program consisted of first an introductory, 2-h session and then a 6-week program of individual learning *via* an app as well as four separate tele-mentoring sessions. In that study, the participants reported improvements with work and their personal lives. Similarly, a study of recruits in the Milwaukee Police Department (Ramey et al., 2017), also with a small sample size (*n* = 34), produced promising results in developing resilience. This program involved a 2-h session, instructions on breathing exercises, as well as four tele-mentor sessions spaced out over approximately 3 months in total.

A study conducted with first responders in Australia (Joyce et al., 2019) who participated in a six-session mindfulness training program (*n* = 143) suggested that “mindfulness-based resilience training delivered in an internet format can create improvements in adaptive resilience and related resources among high-risk workers, such as first responders.”

Finally, two other programs of note are FBI National Academy Associates’ Officer Resilience training (Federal Bureau of Investigation National Academy Associates, n.d.) and the New Jersey Resiliency Program for Law Enforcement (State of New Jersey, 2019). Both have been recently developed, and a review of the literature did produce any published evaluation data on the programs. The FBI program started in 2017, and New Jersey’s program is even newer, beginning in 2019. The only other identified program was developed by the International Association of Chiefs of Police, the U.S. State Department’s Bureau of Justice Assistance, and the University of Pennsylvania’s Positive Psychology Center. They began piloting a resiliency program in multiple jurisdictions across the United States with the intention of a large expansion eventually to other agencies (Bureau of Justice Administration, 2019). Again, no program evaluation data were located.

## The Warr;OR21 Program Explained

As first responder agencies across the globe have embraced the fact that they must work toward the enhancement of the workforce’s well-being, mental health, and resilience, the issue that they are now faced with is how can this be achieved. The first author has developed a program that offers practices that are both practical and sustainable for first responders. The warr;or21 program is a 21-day set of various practices developed based on by previous research that seeks to achieve positive outcomes of increasing first responders’ inner strength, enhancing their resiliency, and in turn, increasing their positive mental health. The first author initially created the program for first responders, but it has also undergone adaptation, making it available to the general public.

Each day over the course of 21 days, first responder participants receive a keyword to guide their practices for the day. Keyword examples include grit, calm, empathy, adapt, and gratitude. The program can work in various ways, but the primary approach is to give a 1.5-h introduction to the program through an in-person workshop and then to conduct the actual program through a private Google Classroom.

During the pilot phase of the program, most cohorts participated through registering in a private Google Classroom. Upon receiving the classroom code, each participant received a daily alert on a mobile device for the day’s practices. One cohort received the daily material on their agency laptops in an electronic folder designated for the program. The majority of cohorts ran Monday through Friday, and thus the weekends did not contain any practices. During the program, participants also shared their thoughts and feedback with fellow participants by posting in the Google Classroom at different points during the 21 days. The program utilizes Google Classroom as it allows participants to access the program material at their own convenience as well as in a safe and private manner since the application had to be downloaded on their personal mobile device.

The daily keyword served as the focus for each day and the daily practices followed the same format: a quote connected to the keyword, 5 min of a controlled breathing practice (instructions are available each day), a short reading detailing the importance of the keyword with links to further reading, a reflection for the day regarding the key points of the article, and an evening gratitude practice. The participants received a small black notebook in which each evening they wrote down their gratitude practice. Practicing gratitude can help to increase positive sleeping habits (Korb, 2015), and specifically writing gratitude practices is especially effective (Mann, 2018; Wong et al., 2018). Research specific to policing has also demonstrated a connection between gratitude with post-traumatic growth (Leppma et al., 2018), higher life satisfaction, and fewer symptoms of depression (McCanlies et al., 2018).

Throughout the 21 days, the warr;or21 program focuses on four pillars of resilience: awareness, wellness, purpose, and positivity. These four, which are unique to this program, draw on previously established neuroscience and resilience research that has used similar terminology that is critical to developing resilience (see, for example, Reivich and Shatte, 2003; Bonanno, 2013; Korb, 2015; Hanson, 2016; Hanson and Hanson, 2018; Margolis and Stoltz, 2018; Southwick and Charney, 2018). The four pillars of resilience are as follows:

1. Awareness: understanding the basics of psychology and its impact on the first responder’s daily life, as well as controlled breathing practices.
2. Wellness: mental and physical health, as both are profoundly connected to each other in relation to developing inner strength and overall positive mental health.
3. Purpose: possessing specific goals for the self and goals that serve a greater good.
4. Positivity: having a positive outlook and relationships, having realistic optimism, and expressing gratitude.

Importantly, warr;or21 takes into account the busy life of a first responder and instead of dismissing that and insisting on daily practices that are time consuming, which would have no practical chance of actually being used, the practices take approximately 15 min per day, with all but the gratitude practice being recommended for completion first thing each morning. The gratitude practice is for completion just prior to going to bed, as among other things, it can assist in positive sleep hygiene (Korb, 2015).

The program intentionally lasts for 21 days, as for any habit to develop and persist, it must occur over a length of time, as repetition is critical for resilience practices to stick (Margolis and Stoltz, 2018). Also, considering the program is only 21 days, it can act as the beginning of a establishing a new perspective and new practices grounded in neuroscience to build one’s inner strength. To sustain this practice, first responder agencies should consider ways, based on the uniqueness of their agency, and means to help to support sustainable personal resiliency practices in its workforce. The keyword and content for each day serves a specific purpose, while at the same time each successive day builds on its predecessors. Again, each keyword draws on previously established research in its relation to mental health and resilience.

Finally, the use of the semicolon is significant. Its inclusion is in part due to inspiration from Project Semicolon (n.d.), an organization working in suicide prevention. Its use of the “;” is to implore those in crisis not to end their lives, but instead to pause (hence the semicolon) and to reach out for help, because their lives are worth living and people do care about them. The warr;or21 program expands on that mindset and reminds participants to pause each day to make sure they allow time to do the daily practices, while also pausing to check in with themselves to see how they are doing.

## Materials and Methods

Participation in the program was voluntary while outreach was conducted by the first author through informal networks and through referrals from previous participants. Evaluation data were collected through a purposeful sample, which is common in qualitative research (Palinkas et al., 2015), across nine different cohorts. The nine cohorts completed the program at various times between September 2019 and March 2020. This selection is consistent with the description and value of a purposeful sample provided by Palinkas et al. (2015), as it involves the identification and selection of individuals have experience in the phenomenon or topic of interest and have communicated their experiences. Ethics approval to conduct this program evaluation was obtained through the Institutional Review Board at Lipscomb University.

In order to conduct a brief evaluation of the program, the data were collected using an online survey. The survey was constructed and administered using the Survey Gizmo platform^1^. Program participants were notified of the 22-question survey through the Google Classroom notification system the day following the completion of the program and then a follow-up reminder was sent again 4 days later^2^. The survey included questions such as demographics, general satisfaction, how useful the program was overall and its specific elements, and program impact on various outcomes, such as resilience, gratitude, stress, and general mental well-being. Survey responses were collected using Likert scale response scales and a number of open-response questions were included in the survey. The open-response questions were used to gain a deeper insight into each participants perspective about the program beyond what Likert scale-responses would be able to provide.

## Program Analysis of Warr;OR21

In order to evaluate the program, the data collected from the survey were analyzed through thematic analysis (Patton, 2002; Braun and Clarke, 2006; Creswell, 2007). This method allows for the experiences of each individual to be highlighted while also allowing for themes to emerge based on the feedback from multiple participants (Creswell, 2007). The participants were able to freely share open-ended feedback during the program while also specific questions in the survey also allowed strategic data to be collected. This is consistent again with thematic analysis and more specifically inductive and theoretical approaches (Thomas, 2003; Braun and Clarke, 2006).

The following analysis is based on responses of 61 individuals who have undertaken the program. The sample included 13 males (21.3%) and 20 females (32.8%), with a significant proportion of the sample not providing a response to this demographic question (28; 46.0%). Of the 61 participants, 36.0% identified as uniformed law enforcement officers, 19.7% were civilians, and 44.3% did not specify employment status.

In terms of general global satisfaction with the program, the evaluation data reveal that program participants held very positive views of the program. Of the sample, 90% were very satisfied (60.0%) or satisfied (30.0%) with the program. Ninety-five percent of participants indicated that they would recommend the program to others in their profession. Comments made by participants included,

“*the best thing I could have done for myself*;”

“*it really helped me become stronger, more positive, and smarter*;”

“*The warr;or21 program is a great tool for anyone but especially in law enforcement*.”

Participants were asked about changes in their attitude toward the war;or21 program at the completion of the program, compared to when they began the program. The majority of participants indicated that their view of the program at the completion of the program was much better (56.7%) or somewhat better (20.0%). Only one program participant indicated a negative view, indicating it was somewhat worse, however, they did not elaborate. Further, 57.1% of the sample indicated that the program exceeded their expectations and 41% indicated it had met expectations. One participant said, *“it gave me a fresh start… it’s a line in the sand for me – myself before the course and after the course.”*

Data were collected relating to the perceived usefulness of the breathing exercises and gratitude practices that were taught as part of the program curriculum and the intention of participants to use these strategies following program completion (see Tables 1 and 2, below). The majority of the program participants found both the breathing exercises and gratitude practices useful.

**Table 1:** Usefulness of program strategies.

Usefulness	Breathing exercises (%)	Gratitude practices (%)
Very useful	61.7	57.1
Useful	26.7	30.4
Neutral	8.3	9
Not useful	3	3.6
Very unuseful	0	0

**Table 2:** Future intention to use program strategies.

Future intentions	Breathing exercises (%)	Gratitude practices (%)
Definitely	68.3	54.1
Probably	25	26.2
Neutral	1.7	14.8
Probably not	5	3.3
Definitely not	0	1.6

Importantly, the majority of program participants indicated that they would continue to use breathing exercises and gratitude practices in the future. A comment made by one participant reflects how the breathing exercise can become integrated into their life, “*…by the time I was half-way through I found myself doing the breathing exercises unconsciously*.”

In terms of program outcomes, participants were asked to rate how much change they perceived they had experienced as a result of undertaken the program. Perceived changes were assessed in respect to resiliency, gratitude, motivation, feelings of calmness, happiness, stress, control of emotions and actions, and overall mental health (see Table 3).

**Table 3:** Perceived changes on key program outcomes.

Program outcome	Rating scale				
	Substantially increased	Somewhat increased	The same	Somewhat decreased	Substantially decreased
Resiliency	40	41.7	18.3	0	0
Gratitude	51.7	35	13.3	0	0
Motivation	35.3	38.2	23.5	3	0
Stress^*^	2.9	14.3	25.7	34.3	22.9
Happiness	41.7	40	18.3	0	0
Calmness	63.2	27.6	9.2	0	0
Control of emotion	26.5	47.1	23.5	3	0
Control of actions	26.5	44.1	26.5	0	3
Feeling out of control^*^	5.7	0.2	31.4	34.3	25.7
Overall mental health	46.7	26.7	26.7	0	0

* reverse coded.

Based on the data, over 50% of the sample perceived substantial increases in their feelings of calmness (63.2%) and gratitude (51.7%). It can be concluded that positive changes were found across all program outcomes for the majority of program participants. When combining responses for “substantial increase” and “somewhat increase” ratings, ratings ranged from 57.2% of the sample (for reductions in stress) up to 90.8% (for calmness).

From the perspective of participants, the least change (feeling the same at the completion of the program compared to the start of the program) included feeling out of control (31.4%), feeling in control of actions (26.5%), stress (25.7%), and motivation (23.5). While 73.4% of the sample perceived positive changes to their overall mental health, just over a quarter of the sample (26.7%) also indicated no change.

## Conclusion

The results of the evaluation of an initial sample of individuals who undertook the warr;or21 program shows promising results. Overall the course was well-received by participants, and they were in the majority satisfied with the course, felt it had met or exceeded their expectations and would recommend the course to others. The specific activities, including breathing and gratitude exercises were both useful for participants while undertaking the program. Perhaps, most importantly, the majority of participants indicated that they intended to keep using the strategies that they had learned during the program. The results of this brief evaluation indicate that the program has most impact on feelings of calmness and gratitude. While the magnitude of change varied across program outcomes, it should be noted that positive changes were experienced by at least 50% of the sample, regardless of which program outcome was studied.

The results of this preliminary evaluation are positive, however, between 18 and 31% of the participants, with exception of calmness and gratitude, did not perceive any change across a number of program outcomes. This does indicate that further evaluation of the program needs to be undertaken to ascertain the reasons why some individuals are not benefiting as much as others. This could include a review of program content and considering the mode of delivery of the content that may be more effective for some than others. Further research is needed to determine whether those that experience less perceived program success have specific characteristics that make them a unique population and what potential adaptation of the program could be undertaken for such groups.

Research has shown that resilience practices, including controlled breathing, gratitude, promoting connectedness, and being able to reframe a situation in a positive perspective can help police officers and other first responders handle stressors associated with their work (McCanlies et al., 2017, 2018; Chopko, 2018; Christopher et al., 2018; Joyce et al., 2018, 2019; Leppma et al., 2018). The warr;or21 program was developed to assist police officers, other first responders, and the general public further develop each of those. Although the data from the program analysis are limited, it has been shown to have a positive impact on many that have participated.

As the program seeks to expand within the first responder community and beyond, it also must be done strategically. For example, incorporating warr;or21 into existing recruit training curriculum should be explored as it can serve numerous benefits aside of developing resiliency. This includes countering the stigma associated with seeking mental health help (Papazoglou and Andersen, 2014).

The warr;or21 program continues to grow, and further evaluation data are necessary to measure its effectiveness and impact. Further evaluations will seek to collect greater information on demographic characteristics to allow for comparative analysis, and the use of pre‐ and post‐ surveys to track changes before and after program participation. The growth of the program since writing this paper and the increase in the number of participants have been substantial. Since the analysis of the initial data for this paper, more than 600 participants have participated in the program. In addition, the program has undergone adaptation to allow non-first responder participants, and this includes people from diverse backgrounds, including physicians, attorneys, clinicians, salespeople, executives, students, retirees, public relations people, and those with other business-related backgrounds. The diversity of the participants includes their locations: Australia, Brazil, Canada, New Zealand, Spain, the United Kingdom, and the United States.

Future development of the program needs to include is the use of a more sophisticated evaluation strategy to better understand the impact of the program. This program data derive from only the responses of participants upon immediately completing the program. Moving forward, evaluation should include, for example, continued follow-up to explore retention of the practices as well as pre‐ and post‐ surveys so that actual changes on key mental health outcomes can be determined, rather than relying on perceptions of participants.

The program continues to develop to capture and analyze the data more effectively. This includes determining which current instruments are most effective, such as possibly the PERMA test (Butler and Kern, 2015), scales that measure coping and flexibility (Bonanno and Pat-Horenczyk, 2011), and various other resilience scales (for a review, see Windle et al., 2011). As first responder agencies continue to explore how to build greater personal resiliency in their workforce, programs, including warr;or21, need to continue to ensure their expansion and progress accompanies rigorous, science-backed methods of evaluation to determine their usefulness.

## Data Availability Statement

Data supporting the conclusions of this article will be made available by the authors, upon request.

## Ethics Statement

The studies involving human participants were reviewed and approved by Institutional Review Board, Lipscomb University. Written informed consent for participation was not required for this study in accordance with the national legislation and the institutional requirements.

## Author Contributions

The two authors contributed to the analysis of the results and to the writing of the manuscript. It is original and has not been submitted elsewhere. All authors contributed to the article and approved the submitted version.

### Conflict of Interest

The authors declare that the research was conducted in the absence of any commercial or financial relationships that could be construed as a potential conflict of interest.

## Footnotes

^1^https://Surveygizmo.com

^2^The survey questions can be accessed by contacting the first author: https://jt2768@cumc.columbia.edu.
